# Management and Predictors of Treatment Failure in Patients with Chemo-Resistant/Relapsed Gestational Trophoblastic Neoplasia with Lung Metastasis

**DOI:** 10.3390/jcm11247270

**Published:** 2022-12-07

**Authors:** Yujia Kong, Weidi Wang, Jinkai Lin, Xirun Wan, Fengzhi Feng, Tong Ren, Jun Zhao, Junjun Yang, Yang Xiang

**Affiliations:** Department of Obstetrics and Gynecology, Peking Union Medical College Hospital, Chinese Academy of Medical Sciences and Peking Union Medical College, National Clinical Research Center for Obstetrics and Gynecologic Diseases, State Key Laboratory of Complex Severe and Rare Diseases, No. 1 Shuaifuyuan Wangfujing, Dongcheng District, Beijing 100730, China

**Keywords:** gestational trophoblastic neoplasia, chemo-resistance, relapse, lung metastasis, salvage chemotherapy, pulmonary resection

## Abstract

The aim of the study was to assess the effectiveness of a combined treatment modality of salvage chemotherapy and pulmonary resection in chemo-resistant/relapsed gestational trophoblastic neoplasia (GTN) with lung metastasis and identify predictors of treatment failure. Data of patients with chemo-resistant/relapsed GTN with lung metastasis who received salvage chemotherapy combined with pulmonary resection were retrospectively analyzed. Among 134 included patients, the number of preoperative chemotherapy regimens ranged from 2–8 (median, 3), and courses ranged from 4–37 (median, 14). Pulmonary lobectomies, segmentectomies, wedge resections, and lobectomies plus wedge resections were performed in 84, 5, 35, and 10 patients, respectively. After completion of treatment, 130 (97.0%) patients achieved complete remission. In the entire cohort, the 5-year overall survival (OS) rate was 87.6%. OS rates were similar between stage III and stage IV disease cohorts (89.4% vs. 75.0%, *p* = 0.137). Preoperative β-human chorionic gonadotropin (β-hCG) levels > 10 IU/L (*p* = 0.027) and number of preoperative chemotherapy regimens > 3 (*p* = 0.018) were predictors of treatment failure. The combined treatment modality of salvage chemotherapy and pulmonary resection is effective in patients with chemo-resistant/relapsed GTN with lung metastasis, improving their prognoses. Patients with preoperative serum β-hCG >10 IU/L and those with >3 chemotherapy regimens preoperatively may not benefit from this multidisciplinary treatment.

## 1. Introduction

Gestational trophoblastic neoplasia (GTN) refers to a group of rare gynecological malignancies caused by abnormal placenta proliferation. GTN consists of invasive moles, choriocarcinomas, placental site trophoblastic tumors (PSTTs), and epithelioid trophoblastic tumors (ETTs) [1]. It is estimated that hydatidiform mole occurs in 0.57 to 2 per 1000 pregnancies worldwide [2]; 15–20% of complete hydatidiform moles and 0.5–5% of partial hydatidiform moles develop into post-molar GTN [3]. Choriocarcinoma occurs in 3 to 23 per 100,000 pregnancies worldwide [4,5,6]. With incidence rates of 0.1–1 per 100,000 pregnancies, PSTT and ETT are rarer [7]. The first-line treatment for GTN is chemotherapy, and approximately 90% of patients are cured owing to its high sensitivity to chemotherapy [3]. However, approximately 17% of patients with GTN show resistance to first-line chemotherapy, and 3.5% of patients relapse after remission [8,9]. In these cases of chemo-resistant/relapsed disease, salvage chemotherapy is required. Apart from salvage chemotherapy, salvage surgeries, such as hysterectomy and resection of metastatic tumors, should not be underestimated [10,11,12,13].

Among patients with chemo-resistant/relapsed GTN, the majority have lung metastasis due to the nature of hematogenous metastasis. Resection of chemo-resistant lesions in the lungs might help these patients achieve remission. Therefore, the most effective treatment strategy for patients with chemo-resistant/relapsed GTN with lung metastasis might be a combined treatment of salvage chemotherapy and pulmonary resection rather than salvage chemotherapy alone. Patients treated with this combined treatment modality achieved a cure rate of 67.8–88.7% in previous small studies [14,15,16,17,18,19]. It has been reported that 40.5% of patients with chemo-resistant/relapsed GTN who underwent pulmonary resection had positive pathological findings [19]. Due to the rarity of GTN, these studies had a small sample size; the efficacy of this combined treatment modality of salvage chemotherapy and pulmonary resection in patients with chemo-resistant/relapsed GTN with lung metastasis needs to be further evaluated. Moreover, a fraction of patients did not have a good response to this multidisciplinary treatment modality. The selection of ideal candidates who can benefit from this combined treatment remains a question.

Thus, this retrospective study was conducted to assess the effectiveness of this combined treatment modality of salvage chemotherapy and pulmonary resection in patients with chemo-resistant/relapsed GTN with lung metastasis and identify predictors of failure to this multidisciplinary treatment.

## 2. Materials and Methods

### 2.1. Patients

**Figure 1:**
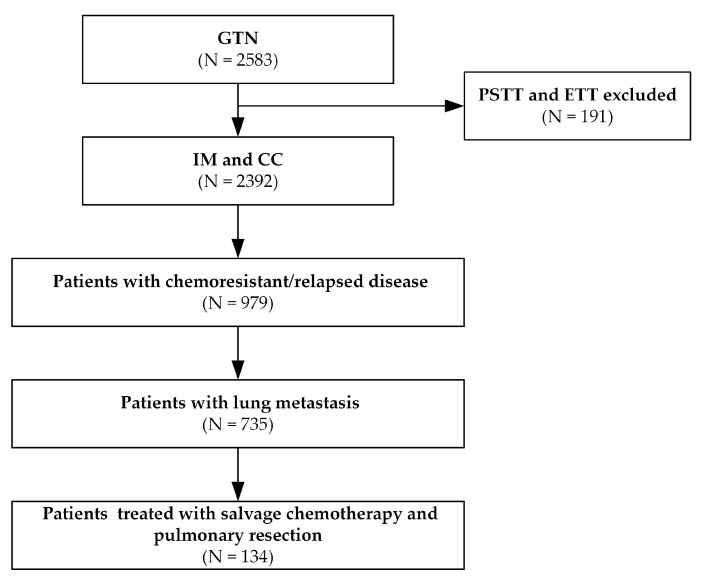
The diagram of patient selection process. GTN, gestational trophoblastic neoplasia; IM, invasive mole; CC, choriocarcinoma; PSTT, placental site trophoblastic tumor; ETT, epithelioid trophoblastic tumor.

The GTN database at Peking Union Medical College Hospital was screened from January 2002 to December 2018. The diagram of patient selection is shown in Figure 1. The following patients were included in this study: (1) patients with chemo-resistant or relapsed GTN; (2) patients who had lung metastasis; and (3) patients who received salvage chemotherapy combined with pulmonary resection. This study excluded patients with PSTT and ETT according to the pathological diagnosis because they are less sensitive to chemotherapy compared with invasive mole and choriocarcinoma. The data were collected through reviewing the medical records and the electronic GTN database, including baseline characteristics, chemotherapy, operative notes, pathological and imaging reports, and laboratory results. All participants signed informed consent before treatment initiation. The ethics committee of Peking Union Medical College Hospital approved this study.

### 2.2. Treatment Protocol

Salvage chemotherapy was chosen according to the patient’s previous failure with chemotherapy. Etoposide, methotrexate, actinomycin D/cyclophosphamide, and vincristine (EMA/CO) was used in patients who had a failed history of floxuridine-based multiagent chemotherapy; floxuridine, actinomycin D, etoposide, and vincristine (FAEV) was used in patients who were resistant to EMA/CO; platinum-contained multiagent chemotherapy was administered in patients with resistance to both FAEV and EMA/CO, including etoposide, methotrexate, actinomycin D/etoposide, cisplatin (EMA/EP) and paclitaxel, cisplatin/paclitaxel, and etoposide (TP/TE). Perioperative chemotherapy was administered to reduce tumor burden and minimize the likelihood of tumor cell metastasis during surgery. In order to reduce the chance of relapse, 3–4 cycles of consolidation chemotherapy were administered.

Pre-treatment evaluations were performed, including magnetic resonance imaging (MRI) of the pelvis, computed tomography (CT) scans of the chest and abdomen, as well as MRI of brain if necessary. Chest CT was repeated before pulmonary resection to identify the site, number, and size of lung metastases. Pulmonary resection was indicated in the following circumstances at our center: (1) patients had good general condition for surgical intervention; (2) patients had chemo-resistant/relapsed GTN; (3) patients had isolated and resectable lung lesions; (4) patients with other metastases outside the lung were also considered for pulmonary resection as long as other metastatic tumors outside the lung had been well controlled after chemotherapy; and (5) preoperative serum β-human chorionic gonadotropin (β-hCG) levels were as low as possible. Pathological reports of surgical specimens were reviewed for all the patients. Positive pathological findings were defined as trophoblastic tumor cells or positive immunohistochemical staining for β-hCG, whereas hemorrhage and necrosis without tumor cells were considered negative pathological findings.

### 2.3. Evaluation

To assess treatment response, serum β-hCG levels were measured once a week. Complete remission (CR) referred to normalization of serum β-hCG levels for at least four consecutive weeks. It was defined as partial remission (PR) if serum β-hCG levels decreased greater than 50%. It was defined as progressive disease (PD) if serum β-hCG levels elevated after salvage chemotherapy combined with surgery. Resistance was diagnosed if serum β-hCG levels plateaued or increased after two cycles of chemotherapy. Relapse was defined as an elevated serum β-hCG one month after CR in the absence of pregnancy. Treatment failure to the combined treatment modality of salvage chemotherapy and pulmonary resection referred to PR or PD or relapse after achieving CR.

### 2.4. Statistical Analysis

In a univariate analysis, correlations between clinical parameters and treatment failure were investigated using Pearson’s chi-square test. In a multivariate analysis, predictors of treatment failure were identified with a logistic regression model. The overall survival (OS) time referred to the time interval from the date of treatment initiation to the date of the last follow-up or death. The 5-year OS rate was calculated based on Kaplan–Meier curve. OS rates among the subgroups were compared using the log-rank test. Statistical Package for Social Sciences (SPSS version 23.0; IBM Corp., Armonk, NY, USA) was used for all analyses. *p* < 0.05 was set as the level of statistical significance.

## 3. Results

### 3.1. Patients Characteristics

During the study period, a total of 134 patients with chemo-resistant/relapsed GTN with lung metastasis received salvage chemotherapy combined with pulmonary resection, of which 44 (32.8%) had chemo-resistant disease, and 90 (67.2%) had relapsed disease. The clinical characteristics of included patients are summarized in Table 1. Of these patients, 14 (10.4%) received primary treatment at our center, and 120 (89.6%) were referred to us after experiencing chemo-resistance or relapse at local hospitals. The median age of these patients was 31 years (range, 20–54 years). Among these patients, 118 (88.1%) patients were at stage III, and 16 (11.9%) were at stage IV based on the 2000 International Federation of Gynecology and Obstetrics (FIGO) staging system. Of the patients with stage IV disease before pulmonary resection, fourteen patients had brain metastasis, one had liver and adrenal gland metastasis, and one had bone metastasis. Prior to pulmonary resection, the maximum diameter of the lung metastases of these patients ranged from 0.5 to 8 cm (median, 1.5 cm). Solitary lung metastases on chest CT were found on 104 (77.6%) patients, while 30 (22.4%) had multiple lung metastases.

### 3.2. Treatment

Among these patients, 102 (76.1%) received FAEV as salvage chemotherapy after experiencing chemo-resistance or relapse, 29 (21.7%) received EMA/CO, and 3 (2.2%) received EMA/EP. Of these patients, 89 (66.4%) achieved normal serum β-hCG levels after single-regimen salvage chemotherapy, while the remaining 45 (33.6%) switched to additional lines of salvage chemotherapy. Overall, the median number of preoperative chemotherapy regimens was three (range, 2–8); 57 (42.5%) patients received >3 lines of chemotherapy preoperatively (including previously failed chemotherapy at local hospitals). The number of preoperative chemotherapy courses ranged as 4–37 (median, 14). The preoperative serum β-hCG level ranged from 0–419 IU/L (median, 1.8 IU/L), with 27 (20.1%) patients that did not achieve normal serum β-hCG levels preoperatively despite usage of multiple lines of salvage chemotherapy.

Pulmonary lobectomies, segmentectomies, wedge resections, and lobectomies plus wedge resections were performed in 84 (62.7%), 5 (3.7%), 35 (26.1%), and 10 (7.5%) patients, respectively. These surgical procedures were performed via thoracotomy (n = 74, 55.2%) and video-assisted thoracoscopic surgery (n = 60, 44.8%). Among these patients, 84 (62.7%) had positive pathological findings postoperatively, while the other 50 (37.3%) only presented with hemorrhage and necrosis without trophoblastic cells. In addition, 58 patients underwent other adjuvant surgical procedures prior to pulmonary resection, including hysterectomy (n = 54), uterine lesion resection (n = 2), craniotomy (n = 1), and resection of tumor in the adrenal gland (n = 1).

### 3.3. Survival

**Figure 2:**
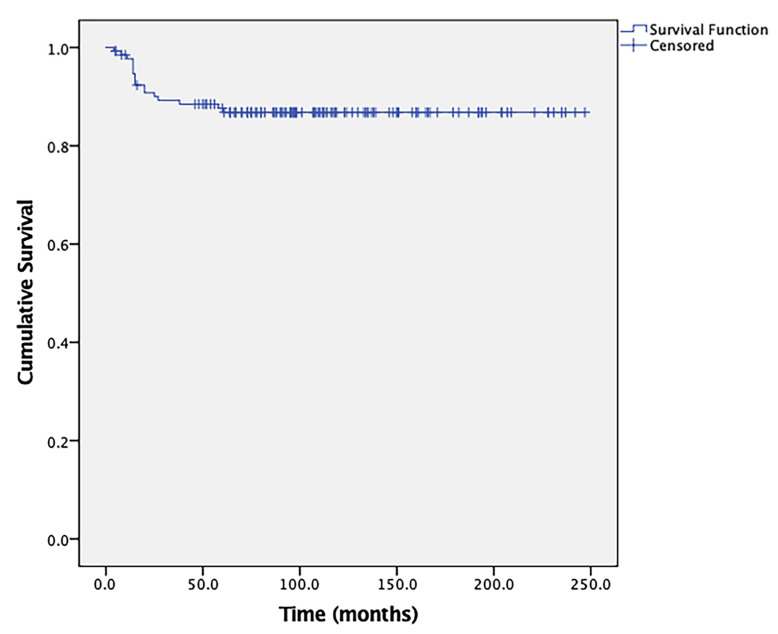
Kaplan–Meier curve showing the overall survival of the study population (N = 134).

After treatment completion, 130 (97.0%) patients achieved CR, 2 (1.5%) achieved PR, and 2 (1.5%) showed PD. Among the patients who achieved CR, 28 (21.5%) experienced relapse. Of the patients who relapsed, three received immune checkpoint inhibitor treatment with pembrolizumab, and all of them re-achieved CR. Aside from four patients who were lost to follow-up, 17 patients died owing to cerebral hernia (n = 4), septic shock (n = 2), respiratory failure (n = 1), and multiple organ failure (n = 10). In the entire cohort, the 5-year OS rate was 87.6% after a median follow-up of 97 months (range, 4–247 months) (Figure 2). Patients with low-risk, high-risk, and ultra-high-risk disease achieved 5-year OS rates of 100%, 88.5%, and 77.8% (*p* = 0.321), respectively. The OS rates of patients with stage III and stage IV disease did not show a statistically significant difference (89.4% vs. 75.0%, *p* = 0.137). Relapse rates between stage III and stage IV disease cohorts were also similar (20.9% vs. 26.7%, *p* = 0.607).

### 3.4. Predictors of Treatment Failure

In this series, a total of 32 (23.9%) patients were considered as treatment failures to salvage chemotherapy combined with pulmonary resection, including 2 with PR, 2 with PD, and 28 with relapse. The treatment failure rates in patients who received FAEV, EMA/CO, and EMA/EP as salvage chemotherapy were 23.5%, 27.6%, and 0, respectively. Several clinical parameters that might predict treatment failure were analyzed. Univariate analysis revealed that multiple lung metastases (*p* = 0.074), preoperative serum β-hCG levels >10 IU/L (*p* = 0.014), and >3 chemotherapy regimens preoperatively (*p* = 0.009) were potential risk factors of treatment failure (Table 2). Multivariate analysis demonstrated that preoperative serum β-hCG levels >10 IU/L (odds ratio (OR): 3.993, 95% confidence interval (CI): 1.170–13.623, *p* = 0.027) and the number of preoperative chemotherapy regimens >3 (OR: 2.801, 95% CI: 1.196–6.559, *p* = 0.018) were predictors of treatment failure to salvage chemotherapy combined with pulmonary resection in chemo-resistant/relapsed GTN (Table 3).

## 4. Discussion

This study confirmed the effectiveness of the multidisciplinary treatment of salvage chemotherapy and pulmonary resection in patients with chemo-resistant/relapsed GTN with lung metastasis. Moreover, this study identified preoperative parameters that may be used to select optimal candidates who can benefit from this combined treatment modality.

Most patients with chemo-resistant/relapsed GTN had lung metastasis, for which the most effective treatment strategy might be salvage chemotherapy combined with resection of chemo-resistant lesions in lung. Several studies have shown the effectiveness of this combined treatment modality in GTN although these studies had small sample sizes. It has been reported that this modality produced CR rates of 67.8–88.7% in GTN [14,15,16,17,18,19]. In this study, 97% of patients with chemo-resistant/relapsed GTN with lung metastasis achieved CR after salvage chemotherapy combined with pulmonary resection. The 5-year OS rate of these patients was 87.6%, which seems to be much higher than the previously reported survival rate of 74.9% in patients with chemo-resistant/relapsed GTN [20]. These results demonstrate that the combined treatment of salvage chemotherapy and pulmonary resection is effective in patients with chemo-resistant/relapsed GTN with lung metastasis and improved their prognoses.

There are several salvage chemotherapy regimens used around the world. For low-risk GTN, actinomycin D or multiagent chemotherapy is used according to serum β-hCG levels. For high-risk GTN, various multiagent salvage chemotherapy regimens are used worldwide, including FAEV, EMA/EP, TP/TE, and high-dose chemotherapy with autologous bone marrow or stem cell transplant. It is uncertain which salvage regimen is the most effective and with minimal toxicity [21,22,23,24,25,26,27]. In our cohort, salvage chemotherapy was chosen based on patient’s previously failed history of chemotherapy. FAEV was the most frequently used salvage chemotherapy at our center, administered in 102 patients who were resistant to EMA/CO. EMA/CO was used in 29 patients who had a failed history of floxuridine-based multiagent chemotherapy, and the remaining 3 patients received EMA/EP due to resistance to both FAEV and EMA/CO. Approximately 66% of patients achieved normal serum β-hCG levels after single-regimen salvage chemotherapy. Overall, salvage chemotherapy should be switched to those chemotherapy regimens with non-cross-resistant drugs in chemo-resistant/relapsed GTN.

Apart from salvage chemotherapy, the role of resection of pulmonary chemo-resistant lesions should be underlined in the management of patients with chemo-resistant/relapsed GTN with lung metastasis. It is generally believed that chemo-resistance or relapse of GTN might be attributed to the presence of chemo-resistant tumor cells or an ineffective concentration of drugs reaching the lesions. Thus, surgical procedures to remove these lesions appear to be the best choice to achieve remission. Our data showed that 62.7% of patients with chemo-resistant/relapsed GTN had positive pathological findings after pulmonary resection, which is consistent with previous research. Cao et al. reported that positive pathological findings after pulmonary resection were found in 40.5% of patients with chemo-resistant/relapsed GTN but only 12% in patients who were sensitive to chemotherapy but had residual pulmonary lesions [19]. These findings suggest the necessity of pulmonary resection to eradicate active tumor cells in the lungs of patients with chemo-resistant/relapsed GTN.

Selecting ideal candidates that can benefit from this combined treatment of salvage chemotherapy and pulmonary resection is another important issue. Previous studies suggested that pulmonary resection should only be performed in patients at stage III without other metastases outside the lung and uterus [14,17,18]. In our current series, 16 patients had stage IV disease before pulmonary resection. There was no statistically significant difference in relapse or survival rates between patients with stage III and those with stage IV disease. Given the small number of patients with stage IV, more evidence is needed to compare the survival of patients with stage III and stage IV who received the combined treatment of pulmonary resection and salvage chemotherapy. However, in our cohort, 75.0% of patients with stage IV were cured after pulmonary resection combined with chemotherapy, which demonstrates that stage IV disease might not be a contraindication for pulmonary resection in patients with chemo-resistant lung lesions. Therefore, comprehensive assessment should be performed prior to pulmonary resection. Patients with stage IV could also be considered for pulmonary resection as long as these metastases outside the lung are well-controlled after chemotherapy. Further studies with larger cohort are needed to test these findings.

Additionally, our data showed that preoperative serum β-hCG levels >10 IU/L and the number of preoperative chemotherapy regimens >3 might predict treatment failure to salvage chemotherapy combined with pulmonary resection. Preoperative serum β-hCG levels have always been associated with outcomes in patients who underwent salvage surgeries; in previous series, the cut-off value ranged from 10–1500 IU/L [18,28]. Additionally, chemo-resistance is the main cause of switch of chemotherapy regimen preoperatively. Patients with more than three lines of chemotherapy preoperatively indicated that they were resistant to multiple chemotherapeutic drugs. These patients were more likely to fail to achieve remission or relapse after remission. Cao et al. also found that patients who received more than four chemotherapy regimens preoperatively had an unfavorable prognosis although this series also included patients who were sensitive to chemotherapy but with residual pulmonary lesions [19]. These data suggest that patients with preoperative serum β-hCG levels >10 IU/L and those who have a failed history of more than three lines of chemotherapy might not benefit from this combined treatment modality.

Novel treatments are urgently needed for those patients who fail to salvage chemotherapy combined with pulmonary resection in chemo-resistant/relapsed GTN. Over the past few years, immune checkpoint blockade has become a successful salvage therapy in GTN [3,29]. Ghorani et al. firstly reported that pembrolizumab was effective in three of four patients with chemo-resistant GTN [30]. A phase 2 trial at our center showed that an objective response rate of 55% was achieved in 20 patients with chemo-resistant/relapsed GTN treated with camrelizumab and apatinib [31]. In our cohort, 32 patients failed to salvage chemotherapy combined with pulmonary resection, 3 of which received pembrolizumab as further salvage therapy; all of them re-achieved CR. Therefore, immune checkpoint inhibitors might be another option for patients who might not benefit from salvage chemotherapy combined with pulmonary resection in chemo-resistant/relapsed GTN.

This study had some limitations. Firstly, we could only conduct a retrospective study because of the rarity of GTN; thus, patient selection and recall biases might have been introduced. In addition, we did not compare the survival of patients with chemo-resistant/relapsed GTN with pulmonary resection and those without. Hence, it is difficult to evaluate how much benefit these patients could obtain from this surgical procedure. It should be noted that a comparison between the two groups is challenging because very few patients with pulmonary chemo-resistant disease did not undergo pulmonary resection.

## 5. Conclusions

The combined treatment modality of salvage chemotherapy and pulmonary resection is effective in patients with chemo-resistant/relapsed GTN with lung metastasis, improving the prognoses of these patients. Stage IV disease might not be a contraindication for pulmonary resection in patients with chemo-resistant lung lesions. Salvage pulmonary resection could also be considered in these patients with comprehensive assessment preoperatively as long as the metastatic tumors outside the lung are well-controlled after chemotherapy. More evidence is needed to test these findings. Patients with preoperative serum β-hCG levels greater than 10 IU/L and those who have a failed history of more than three lines of chemotherapy might not benefit from salvage chemotherapy combined with pulmonary resection. Immune checkpoint inhibitors might be another option as salvage therapy for these patients who failed to this combined treatment.

## Figures and Tables

**Table 1:** Clinical characteristics of the overall study population (N = 134).

Factors	No.	%
Age (years)		
<40	110	82.1
≥40	24	17.9
Antecedent pregnancy		
Mole	52	38.8
Abortion	32	23.9
Term	50	37.3
Interval from index pregnancy to chemotherapy (months)		
<4	2	1.5
4–6	4	3.0
7–12	12	8.9
>12	116	86.6
Pre-treatment serum β-hCG (IU/L)		
<10^3^	85	63.4
10^3^–10^4^	34	25.4
10^4^–10^5^	13	9.7
>10^5^	2	1.5
FIGO stage		
Ⅲ	118	88.1
Ⅳ	16	11.9
WHO prognostic score		
≤6 (low risk)	7	5.2
7–12 (high risk)	108	80.6
≥13 (ultra-high risk)	19	14.2
Maximum diameter of lung metastasis (cm)		
<2	96	71.6
2–3	23	17.2
>3	13	9.7
NA	2	1.5
Site of lung metastasis		
Unilateral	129	96.3
Bilateral	5	3.7
No. of lung metastasis		
Solitary	104	77.6
Multiple	30	22.4

Abbreviations: β-hCG, β-human chorionic gonadotropin; FIGO, International Federation of Gynecology and Obstetrics; WHO, World Health Organization; NA, not available.

**Table 2:** Univariate analysis of factors predicting treatment failure to salvage chemotherapy combined with pulmonary resection in the overall study population (N = 134).

Factors	Treatment Failure Rate (%)	*p*
Age (years)		
<40	23.6	0.537
≥40	25.0	
Antecedent pregnancy		
Mole	21.2	0.678
Abortion or term	25.6	
FIGO stage		
Ⅲ	28.2	0.176
Ⅳ	31.3	
WHO prognostic score		
≤6 (low risk)	14.3	0.928
7–12 (high risk)	24.1	
≥13 (ultra-high risk)	26.3	
No. of lung metastasis		
Solitary	21.2	0.074
Multiple	33.3	
Maximum diameter of lung metastasis (cm)		
≤3	22.7	0.362
>3	30.8	
Pathological finding		
Positive	22.6	0.404
Negative	26.0	
Preoperative serum β-hCG (IU/L)		
≤10	20.7	0.014
>10	53.8	
No. of preoperative chemotherapy regimens		
≤3	15.6	0.009
>3	35.1	
Operative approach		
Thoracotomy	24.3	0.894
VATS	23.3	

Abbreviations: FIGO, International Federation of Gynecology and Obstetrics; WHO, World Health Organization; β-hCG, β-human chorionic gonadotropin; VATS, video-assisted thoracoscopic surgery.

**Table 3:** Multivariate analysis of factors predicting treatment failure to salvage chemotherapy combined with pulmonary resection in the overall study population (N = 134).

Factors	Odds Ratio	95% Confidence Interval	*p*
No. of lung metastasis			
Multiple vs. solitary	2.052	0.794–5.304	0.138
Preoperative serum β-hCG (IU/L)			
>10 vs. ≤10	3.993	1.170–13.623	0.027
No. of preoperative chemotherapy regimens			
>3 vs. ≤3	2.801	1.196–6.559	0.018

Abbreviations: β-hCG, β-human chorionic gonadotropin.

## Data Availability

The data presented in this study are available on request from the corresponding author.
